# Identification and Re-Evaluation of Freshwater Catfishes through DNA Barcoding

**DOI:** 10.1371/journal.pone.0049950

**Published:** 2012-11-15

**Authors:** Maloyjo J. Bhattacharjee, Boni A. Laskar, Bishal Dhar, Sankar K. Ghosh

**Affiliations:** Department of Biotechnology, Assam University, Assam, India; University of Veterinary Medicine Hanover, Germany

## Abstract

**Background:**

Catfishes are globally demanded as human food, angling sport and aquariums keeping thus are highly exploited all over the world. North-East India possess high abundance of catfishes and are equally exploited through decades. The strategies for conservation necessitate understanding the actual species composition, which is hampered due to sporadic descriptions of the species through traditional taxonomy. Therefore, actual catfish diversity in this region is important to be studied through the combined approach of morphological and molecular technique of DNA barcoding.

**Methodology/Principal Findings:**

Altogether 75 native catfish specimens were collected from across the North-East India and their morphological features were compared with the taxonomic keys. The detailed taxonomic study identified 25 species belonging to 17 genera and 9 families. The cytochrome oxidase c subunit-I gene fragment were then sequenced from the samples in accordance with the standard DNA barcoding protocols. The sequences were compared with public databases, viz., GenBank and BOLD. Sequences developed in the current study and from databases of the same and related taxa were analyzed to calculate the congeneric and conspecific genetic divergences using Kimura 2-parameter distance model, and a Neighbor Joining tree was created using software MEGA5.1. The DNA barcoding approach delineated 21 distinct species showing 4.33 folds of difference between the nearest congeners. Four species, viz., *Amblyceps apangi*, *Glyptothorax telchitta*, *G. trilineatus* and *Erethistes pusillus*, showed high conspecific divergence; hence their identification through molecular approach remained inconclusive. On the other hand, the database sequences for three species, viz., *Mystus horai, Bagarius yarrelli* and *Clarias batrachus*, appeared mislabeled.

**Conclusion:**

The efficiency of DNA barcoding was reaffirmed from its success by easily identifying the major share (84%) of the studied catfish into 21 distinct species. The study contributed 27 new barcodes for 7 species and confirmed the range expansion of 2 important species in NE India.

## Introduction

Catfishes are members of the order Siluriformes (Actinopterygii) and inhabit inland and marine ecosystems. They are generally bottom dwellers and feed upon almost any kind of plant or animal matters, hence, play an important role in transferring energy throughout the food web [1]. Most of the species hold demand all over the world, including North-East (NE) India, as human food and aquarium keeping [2]. However, due to pressure from unregulated harvest for commercial sale along with other anthropogenic and environmental threats in NE India, some of the native catfish species have become threatened (www.iucnredlist.org). NE India is rich in biodiversity and shares two of the Biodiversity Hotspots in the world, viz., the Eastern Himalaya and the Indo-Burma [3]. The region is bestowed with numerous water bodies of diverse nature and is home to around 267 species of fishes, including many endemic catfishes such as *Amblyceps apangi*, *Amblyceps arunachalensis*, *Glyptothorax striatus* [4]. The inventories of fishes from this region were plethoric and entirely based on conventional taxonomy. Due to adherent impediments with traditional taxonomy [5], a few species once claimed new are remarked to be not valid. Instead, many cases of synonym species have been uncovered and await taxonomic revision. For example, the occurrence of *M. vittatus* in NE has been debated repeatedly [6], [7], [8] and is a great concern for the systematic. Moreover, taxonomic confusions exist with some other species of the genera *Sperata, Ompok, Eutropiichthys, Clupisoma, Gagata* and *Nangra*. The congeners of *Sperata* are distinguishable by either round or spatula-shaped snout or length of maxillary barbells that either extends to base of caudal fin or no further than pelvic fins. The congeners of *Eutropiichthys* are differentiable based on length of maxillary barbells and number of fin rays. Since all such characters are prominent only in adults hence the specimens at early stage are difficult to identify [6]. *Ompok bimaculatus* has been considered “restricted in southern India” and its conspecificity throughout the Indian subcontinent was remarked to be doubtful. Rather, the populations of the species from different areas of the subcontinent were assumed to be representing different species [9], [10]. Nevertheless, the congeners of *Amblyceps* from NE India were often synonymized with *Amblyceps mangois* [11]. *Pterocryptus indicus* was often described endemic in NE India but mostly remarked doubtful about its validity [12]. Many such cases are due to unresolved issues related to proper documentation of catfish diversity in NE India. Indeed, the earlier inventories seem to be non-exclusive and sporadic and a correct checklist of catfish diversity in the region is unavailable. Therefore, evaluation of actual catfish diversity using molecular tools is important to resolve the species and develop strategies for conservation of threatened taxa in the region. It is expected that the perplexity in identification of many catfish species of the region can be resolved and made easier through the intervention of the advanced DNA barcode based species identification technique.

**Table 1:** Summary of identification based on each species consensus barcoded sequence using BLASTN search from GenBank and BOLD Identification System (BOLD-IDS).

Sl. No.	Studied species	Species match by name		% Similarity	
		GenBank (BLASTN)	BOLD-IDS	GenBank (BLASTN)	BOLD-IDS
1.	*Rita rita* (1)	*Rita rita*	*Rita rita*	99	100
2.	*Mystus bleekeri* (8)	*Mystus bocourti*	*No match*	88	No match
3.	*M. cavasius* (4)	*Mystus oculatus*	*No match*	89	No match
4.	***M. vittatus*** ** (7)**	*Mystus vittatus*	*Mystus vittatus*	99	99.83
		***Mystus horai***	***Mystus horai***	**99**	**99.64**
5.	*Sperata aor* (3)	*Sperata aor*	*Sperata aor*	100	100
6.	*Hemibagrus menoda* (2*)*	*Sperata aor*	*No match*	86	No match
7.	***Bagarius bagarius*** ** (3)**	*Bagarius bagarius*	*Bagarius bagarius*	100	100
		***Bagarius yarrelli***	***Bagarius yarrelli***	**100**	**100**
		*Bagarius yarrelli*		91	
8.	*Gagata cenia* (6)	*Gagata cenia*	*Gagata cenia*	99	99.5
9.	*Gagata sexualis* (2)	*Gagata sexualis*	*Gagata sexualis*	99	99
10.	***Glyptothorax telchitta*** ** (3)**	***Glyptothorax telchitta***	*No match*	**93**	No match
11.	*G. striatus* (1)	*Glyptothorax striatus*	*Glyptothorax striatus*	97	97.6
12.	***G. trilineatus*** ** (1)**	***Glyptothorax trilineatus***	*No match*	**96**	No match
13.	*Sisor rabdophorus* (4)	*Sisor rabdophorus*	*Sisor rabdophorus*	100	100
14.	*Ailia coila* (3)	*Ailia coila*	*Ailia coila*	99	99.67
15.	*Clupisoma garua* (1)	*Laides hexanema*	*No match*	91	No match
16.	*Eutropiichthys murius* (3)	*Pangasius larnaudii*	*No match*	86	No match
17.	*E. vacha* (7)	*Laides hexanema*	*No match*	89	No match
18.	*Ompok bimaculatus* (2)	*Ompok bimaculatus*	*Ompok bimaculatus*	99	99.84
19.	*O. pabo* (2)	*O. pabo*	*O. pabo*	99	99.33
20.	*Wallago attu* (2)	*Wallago attu*	*Wallago attu*	100	100
21.	***Clarias batrachus*** ** (2)**	*Clarias batrachus*	*Clarias batrachus*	98	98.64
		***Clarias batrachus***		**90**	
22.	*Heteropneustes fossilis* (3)	*Heteropneustes fossilis*	*No match*	100	No match
23.	*Erethistes pusillus* (2)	*Erethistes pusillus*	*No match*	93	No match
24.	***Amblyceps apangi*** ** (1)**	*Amblyceps apangi*	*No match*	95	No match
		***Amblyecps apangi***		**88**	
25.	*Olyra longicaudata* (1)	*Amblyceps mucronatum*	*No match*	90	No match

• Similarity description used in the study- 97%–100%– significant, 92%–96%– moderate, ≤91%– insignificant.

• Bolded words correspond to problematic identification of species in the present study using either one or both the databases. Details are further discussed in the text.

• Numbers in brackets indicate the number of individual sequences of each species.

The DNA barcoding concept has been launched as a rapid, accurate, automatable, and globally accessible procedure for species delimitation and identification [13]. The effectiveness of this method relies on the relatively conserved stretch of approximately 655 nucleotides of the mitochondrial cytochrome oxidase c subunit-I (COI) gene. Based on the nucleotide sequences, accurate identification of organisms at the species level is reasonably straightforward and has been applied to numerous animal taxa [14], [15], [16], [17]. The DNA barcode reference library is rapidly growing by the contributions of the global community in the Barcode of Life Data Systems (BOLD) [18]. With the glory as an attractive species identifier in fish biodiversity research [19], the application of DNA barcoding has recently reflected 28% increase of North American freshwater fish diversity [20]. The technique further bears application in monitoring fish products for health safety [21], [22] and in regulating the exploitation of fish species under aquarium trade [23], [24], [25].

**Figure 1:**
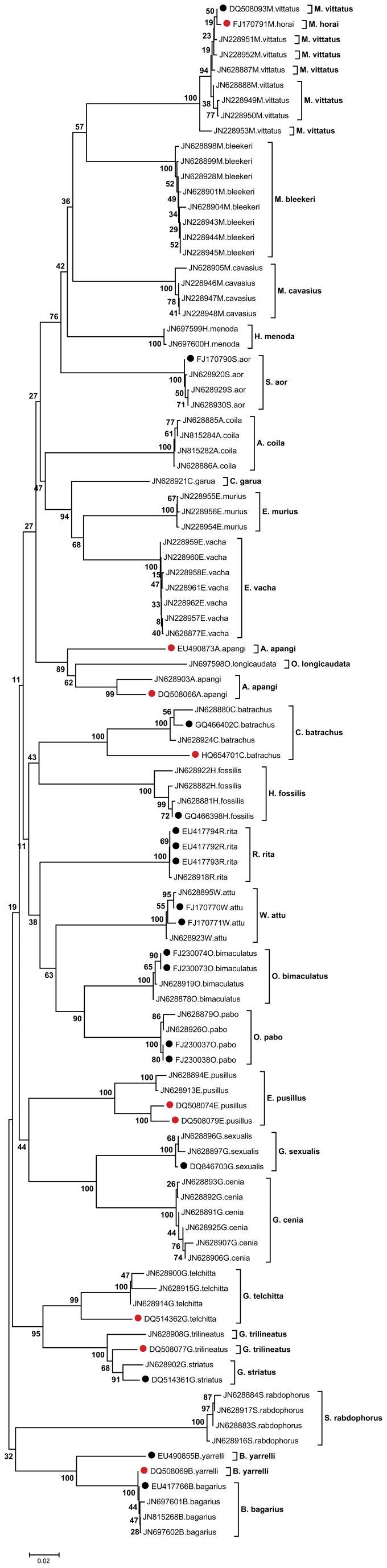
Neighbor joining (NJ) tree developed using K2P distance among 101 CO1 sequences. Notable anomalies in clustering are shown by 4 species {*Mystus horai* (accession number FJ170791), *Bagarius yarrelli* (accession number DQ508069), *Clarius batrachus* (accession number HQ654701) and *Amblyceps apangi* (accession number EU490873). Deep conspecific divergences are shown by 3 species (*Glyptothorax trilineatus, Glyptothorax telchitta* and *Erethistes pusillus*). · The numbers at the nodes are bootstrap values based on 1000 replications. · Specimen GenBank accession number and species name are shown for each taxon. · Red and black dots correspond to the sequences acquired from database. Red dot alone corresponds to the cases of abnormal clustering and deep conspecific divergence.

DNA barcoding technique was adopted in NE India to study the actual diversity of catfishes inhabiting in the region. This will also enrich the database by contributing both new barcodes and replica of existing barcodes thereby enabling the evaluation of taxonomic status of the native catfish diversity in NE India. Here we studied the first DNA barcode based taxonomic resolution of freshwater catfishes from NE India to resolve key areas of doubt arising from morphological taxonomy. This investigation not only prove the potential use of DNA barcoding as a tool to aid traditional taxonomy of freshwater catfish but also will help further in easy identification of the studied species from any of their body parts and at any stage of life. Nevertheless the sequences generated from this study would be accessible to establish the conspecificity of NE Indian catfish with other geographical location and vice versa.

**Figure 2:**
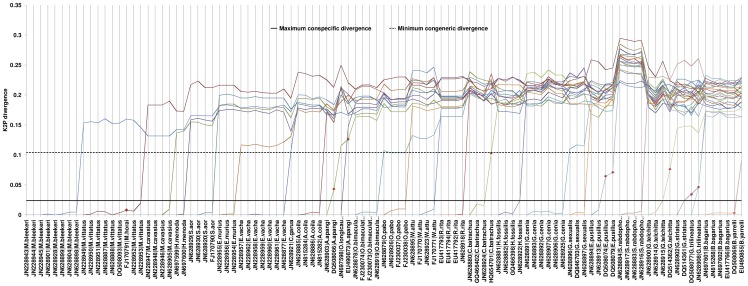
Congeneric and conspecific K2P divergence for 101 sequences of 27 catfish species. The maximum conspecific divergence (0.024, black solid line) and minimum congeneric divergence (0.104, black dotted line) represent the threshold level of conspecific and congeneric divergence respectively. Most of the studied species (21) obeyed the thresholds and are readily delineated showing barcoding gap of 4.33 or above. Sequences of species like *M. horai* (accession number FJ170791), *B. yarrelli* (accession number DG508069), *A. apangi* (accession numbers EU490873 and DQ508066), *C. batrachus* (accession number HQ654701), *E. pusillus* (accession numbers DQ508074 and DQ508079), *G. telchitta* (accession number DQ514362), *G. trilineatus* (accession number DQ508077) did not obey the thresholds and are thus ambiguous (shown in red dots). *G. striatus* with two sequences obeyed the threshold of maximum conspecific divergence and minimum congeneric divergence with all congeners except *G. trilineatus.* · In the X-axis the specimens involved in this study were plotted and marked as, GenBank accession number|species name.

## Materials and Methods

### Sample collection

The native catfishes were collected from different natural water bodies during different seasons of the year from random geographical locations within the NE region of India (28°09^/^N 97°24^/^E on the East to 27°49^/^N 88°15^/^E on the West, and 29°18^/^N 96°04^/^E on the North to 92°59^/^E on the South). Fresh specimens were spot examined for specific morphological characters that define the catfish and sampled from different wild habitats as and when caught by the professional fishers. Each of the catch was investigated by an experienced fish taxonomist to ensure correct sampling and labeling. Upon every spate of collection, the tissue samples from each of the specimens were collected aseptically and preserved in 90% ethanol. Major taxonomic keys of each of the fish specimens were noted and measurements were taken using a digital caliper. Species level identification of the specimens was confirmed by comparing with the described characters and the taxonomic keys available in the leading taxonomic guides of the fishes in India [6], [26]. However, the nomenclature of species follows the Catalogue of Fishes [27]. The comparisons of the observed characters in respect of each species with their described characters along with the particular dispute are presented in Table S1. Altogether 75 fish specimens belonging to 25 species within 17 genera and 9 families were collected and included in this study. All the voucher specimens have been deposited in the Department of Biotechnology, Assam University, Silchar. The specimen information, IUCN Red list status and distribution of the studied species are given in Table S2.

Since the studied fishes were routine caught by the professional fishers for sale hence no permission was required for their sampling.

### DNA extraction

20 mg of anal fin tissue was taken aseptically and dissolved in 500 μL of TES buffer (50 mM Tris HCl, 25 mM EDTA and 150 mM NaCl) in a microcentrifuge tube. The extraction of DNA was performed with Phenol-Chloroform-Isoamylalcohol method [28].

### PCR amplification and purification

The COI gene (655 bp) was amplified using the set of published primers [29] as follows. FishF1-5^/^ TCAACCAACCACAAAGACATTGGCAC 3^/^ and FishR1-5^/^ TAGACTTCTGGGTGGCCAAAGAATCA 3^/^ in a Veriti Mastercycler (Applied Biosystems Inc., CA, USA). The amplification reactions were performed in a total volume of 25 µl comprising 1X PCR buffer, 2 mM MgCl_2_, 10 pmol of each primer, 0.25 mM of each dNTPs, 0.25 U of high-fidelity Taq polymerase (Applied Biosystems Inc., CA, USA) and 100 ng of DNA template. The thermal profile of the PCR reaction was as follows: An initial denaturation at 94°C for 2 minutes, 30 cycles at denaturation temperature of 94°C for 45 seconds, annealing temperature of 50°C for 45 seconds and elongation temperature of 72°C for 1 minute, and concluded with a final elongation step at 72°C for 8 minutes followed by a hold at 4°C. The PCR-amplified products were analyzed in 1% agarose gels containing ethidium bromide staining (10 mg/ml) and the single uniform band was then purified using QIAquick^R^ Gel extraction kit (QIAGEN, USA), following manufacturer's instructions. The amplicons were bidirectionally sequenced in an automated DNA sequencer (ABI 3500, Applied Biosystems Inc., CA, USA), through the best known service of GCC Biotech India Pvt. Ltd. (Kolkata, India). The COI amplicons were recovered from all the collected specimens.

### Sequence quality control measures

Both the PCR amplified products and their corresponding DNA sequences were larger than 600 bp that assured no NUMTs being amplified as the limit of NUMT hardly reaches 600 bp [30]. Ends of the noisy sequences were trimmed and more than 600 bp sequences were used for the final analysis (except in four cases, accession numbers JN697602, JN628915, JN628929 and JN628930). For each sample two chromatograms that represent sequences of both the strands of DNA were obtained. BLASTN [31] program was used to compare the sequences from the two chromatograms, and the fragment of the two sequences showing 100% alignment with no gap or indels (insertion/deletions) was selected. In case of any discrepancy, both the sequences were reviewed and quality value of the sequence was considered to determine the most likely nucleotide using the software SeqScanner Version 1.0 (Applied Biosystems Inc., CA, USA). In most cases, the sequence quality values were above 50. The selected fragments of the sequences for all the specimens were aligned using ClustalX software [32] and found no indels in any of the sequences. Finally, each of the sequences were subjected to BLASTN searches at the National Centre for Biotechnology Information [31], that showed alignment with the partial coding sequence of fish mitochondrial COI gene without any indels. The sequences were translated using the online software ORF finder (http://www.ncbi.nlm.nih.gov/gorf/gorf.html) and aligned through BLASTP [31] that revealed coherent partial amino acid codes with fish mitochondrial COI gene frame without any stop codon. Therefore, it was confirmed that the generated sequences were fragments of mitochondrial COI gene. All the analyzed sequences were then deposited in GenBank and received valid accession numbers (Table S1). The sequences were also submitted by creating a FISH-BOL project in BOLD in the code name of ‘CFISH’ entitled “DNA barcoding of freshwater catfishes of Northeast India”.

### Data analysis

The BOLD provided 778 COI sequences for catfish (accessed on 17 November, 2011) among them the database sequences of the same and/or related taxa are used in association with developed sequences for evaluating the taxonomic status of our target species. The total dataset included 101 COI barcode sequences for 27 catfish species among which 75 sequences belonging to 25 species were developed de-novo, and 26 sequences representing same and related taxa were acquired from GenBank only because there were no additional sequences available in BOLD other than those mined from GenBank source. Geographical information and GenBank accession numbers of the developed as well as acquired database sequences are given in Table S2. The sequence similarity search for species identification was done in two public databases, viz., BOLD and GenBank. The highest percent pairwise identity for each sequence blasted (BLASTN) at NCBI were compared with the percent similarity scores of the same sequence within the BOLD-IDS (BOLD Identification System) [18]. The query species that matched either with the same or different species in the databases has been termed as ‘specific’ or ‘non-specific’ respectively. The similarity range of 97%–100%, 92%–96% and ≤91% between the query and the database sequence have been expressed as significant, moderate and insignificant respectively. Kimura 2-parameter (K2P) congeneric and conspecific variation [33] and Neighbor Joining (NJ) tree construction were done using the computer program MEGA Version 5 [34]. Maximum conspecific and minimum congeneric divergences have been determined considering the sequences showing cohesive NJ clustering within a species and remained distinct from other species. The number of times the minimum congeneric divergence differs from the maximum conspecific divergence is the lowest divergence between congeners and has been assumed to be the threshold level of species delineation and thereby considered as a barcoding gap in this study.

## Results

Comprehensive species identification of the studied catfishes based on BOLD and GenBank databases is depicted in Table 1. The study helped in straightforward identification of 10 species that showed significant species specific similarities in both the databases. The species are *Sperata aor, Sisor rabdophorus*, *Wallago attu*, *Gagata sexualis*, *Rita rita*, *Gagata cenia*, *Glyptothorax striatus*, *Ailia coila*, *Ompok bimaculatus* and *Ompok pabo*. GenBank sequences showed moderate species specific similarity for both *Glyptothorax telchitta* and *Erethistes pusillus* at 93%, *G. trilineatus* at 96% and significant species specific similarity for *Heteropneustes fossilis* at 100%. It also showed insignificant non-specific similarity (≤91%) for seven species, viz., *Mystus bleekeri*, *M. cavasius*, *Hemibagrus menoda*, *Clupisoma garua*, *Eutropiichthys murius*, *E. vacha* and *Olyra longicaudata.* Both the databases *s*howed significant species specific similarity (≥98%) for *M. vittatus, B. Bagarius* and *Clarias batrachus*, as well as significant non-specific similarity (≥99%) for *M. vittatus* (query) with *M. horai* (database accession number FJ170791), and *B. bagarius* (query) with *B. yarrelli* (database accession number DQ508069). GenBank alone showed insignificant species specific similarity (90%) for *C. batrachus* with a database accession number HQ654701. GenBank concurrently showed moderate species specific similarity (95%) and insignificant species specific similarity (88%) for *Amblyceps apangi* with database accession numbers DQ508066 and EU490873 respectively.

The similarity search result thereby confirmed definitive identity showing significant species specific match in GenBank and BOLD for 11 species, viz., *R. rita*, *S. aor*, *G. cenia*, *G. sexualis*, *G. striatus*, *S. rabdophorus*, *A. coila*, *O. bimaculatus*, *O. pabo, W. attu* and *H. fossilis*. *H. fossilis* latter was identified by GenBank alone. The rest of the studied species (14) showed ambiguous match categories, like, 1) significant but equally species specific and non-specific (e.g. *M. vittatus* and *B. Bagarius*), 2) species specific but equally significant and insignificant (e.g. *C. batrachus*), 3) species specific but moderate (e.g. *G. telchitta, G. trilineatus, E. pusillus* and *A. apangi*), 4) species specific but insignificant (e.g. *A. apangi* accession number EU490873), and 5) non-specific and insignificant (e.g. *M. bleekeri*, *M. cavasius*, *H. menoda*, *C. garua*, *E. murius*, *E. vacha* and *O. longicaudata*).

The Neighbor Joining (NJ) cluster analysis (Figure 1) revealed straight forward identification showing either a single or distinct cluster of individual(s) for 18 of our studied species. These include 11 accurately identified species which showed significant species specific similarity and 7 species which showed insignificant non-specific match (in parity with ambiguous match category-5). However, ambiguities persisted for 7 other species and showed three distinct patterns: 1) same and different named-species clustered together (in parity with ambiguous match category-1, e.g., all sequences of *M. vittatus* clustered with *M. horai* (accession number FJ170791) and all sequences of *B. bagarius* clustered with *B. yarrelli* (accession number DQ508069), 2) same named-species clustered both jointly and distinctly (in parity with ambiguous match category-2, e.g., *C. batrachus* of accession number GQ466402 and HQ654701), and same named-species clustered only distinctly (in parity with ambiguous match category-4, e.g., *A. apangi* of accession number EU490873), and 3) high range of clustering differences with conspecific query sequences (in parity with ambiguous match category-3, e.g., *G. telchitta* of accession number DQ514362, *G. trilineatus* of accession number DQ508077, *E. pusillus* of accession number DQ508074 and DQ508079, and *A. apangi* of accession number DQ508066).

The minimum congeneric and maximum conspecific K2P divergences were determined to be 0.104 and 0.024 respectively and presented in Figure 2. Based on these divergence values, a 4.33 folds barcoding gap was calculated. 72% (18) of the studied species identified through the NJ clustering have also been easily delineated following the barcoding gap. Few database sequences of the same and/or related species have not obeyed this gap and hence designated as ambiguous. For example, 1) a few congeneric sequences merged within the range of conspecific divergence (e.g., *M. horai* of accession number FJ170791 and *B. yarrelli* of accession number DQ506089) and vice versa (e.g., *C. batrachus* of accession number HQ654701 and *A. apangi* of accession number EU490873), 2) a few individual sequences were widely dispersed from their conspecific sequences and did not reach the congeneric threshold divergence (e.g., *G. trilineatus* of accession number DQ508077, *G. telchitta* of accession number DQ514362, *E. pusillus* of accession numbers DQ508074 and DQ508079 and *A. apangi* of accession number DQ508066), and finally 3) congeneric distances of *G. striatus* with G. *trilineatus* have remained much below the congeneric threshold divergence.

## Discussion

This study of identification of catfishes from NE India was based on the morphological investigation followed by DNA barcoding approach. The morphological study of the specimens has raised a few questions on the observed features versus the described features. In a few cases, morphological species keys were difficult to discern. Moreover, disparities relating to the species keys were observed in a few cases between the two leading taxonomic guide books of fishes in India (Table S1). The DNA barcoding approach resolved some identification issues and explained the actual species composition in the region.

Among the 25 studied catfish species, the similarity search approach revealed two straightforward cases for 18 species. Firstly, 11 species, viz., *R. rita*, *S. aor*, *G. cenia*, *G. sexualis*, *G. striatus*, *S. rabdophorus*, *A. coila*, *O. bimaculatus*, *O. pabo*, *W. attu* and *H. fossilis*, showed significant species specific similarity in the range of 97%–100%, and were readily identified as true species. Secondly, seven species, viz., *M. bleekeri*, *M. cavasius*, *H. menoda*, *C. garua*, *E. murius*, *E. vacha* and *O. longicaudata*, showed insignificant non-specific similarity (≤91%) in GenBank and no match in BOLD. This has reflected the lack of barcode reference data for these species in both the databases. However, all the sequences of the above mentioned 18 species showed conspecific NJ clustering by the specimens within each species having well supported bootstrap proportion (>95%) [35]. Further, all the 18 species were definitely delineated considering barcoding gap principle (Figure 2) and identified as true species based on the combined approaches, including 7 species whose barcode data were not available in the databases previously. So, the study contributed new barcode data in the global database for those seven species. Range expansion of *O. bimaculatus* in NE India was also evident from this study through observed high conspecificity among the queries and database sequences from the Ganga basin (FJ230073–4) in India. We concentrated to delineate the species based on the threshold level of species divergence taking the maximum conspecific versus minimum congeneric divergence into account rather than considering the conventional mean value of congeneric and conspecific divergence. This has led to the reflection of the lowest barcoding gap of 4.33 folds compared to the previous DNA barcoding studies of fishes [23], [29], [36], [37], [38].

While morphological identification were convincing up to species-level for all the studied species, DNA barcoding even remained inconclusive for 7 species (viz., *M. vittatus*, *B*. *bagarius*, *C. batrachus*, *A. apangi*, *G. trilineatus*, *G. telchitta*, and *E. pusillus*), at its first hand approach. Combined approach has confirmed that database sequence of *M. horai* and *M. vittatus* are conspecific. Moreover, the query sequences of *M. vittatus* showed conspecificity with a same named database sequence of accession number DQ508093. Previous morphological studies have already raised doubtfulness on the taxonomic validity of *M. horai* and the species was remarked to be not recorded from any other location than its type locality (in Indus drainage) [27], [39]. Since the molecular evidences also reckon the previous morphological debate, hence the study tentatively considered *M. horai* as a synonym of *M. vittatus*. As such the study identified *M. vittatus* being a true species and recognized its range expansion in NE India. Therefore, the study resolved the debate surrounding the existence of *M. vittatus* in NE India [7], [8]. Again, one of the two database sequences of *B. yarrelli* (accession number DQ508069) revealed conspecific divergence with *B. bagarius* while the other sequence (accession number EU490855) maintained congeneric divergence with the same. This reflected that the former sequence of *B. yarrelli* is mislabeled in the database. This study thus confirmed both *B. bagarius* and *B. yarrelli* being true species and met with the previous argument [40]. In case of *C. batrachus*, a single database sequence (accession number HQ654701) showed congeneric divergence with other conspecific sequences. On the other hand, the queries as well as the other database sequences of *C. batrachus* showed conspecificity. This confirmed *C. batrachus* to be a true species and indicated a clear mislabeling of the said sequence in the database. Similar cases of mislabeling have also been reported earlier [22]. Ignoring the few mislabeled database sequences, the identification of above three species was confirmed.

In another case, one of the two database sequences of *Amblyceps apangi* (accession number EU490873) was identified to be a distinct congener of the query sequence. This may be again a case of mislabeling because the two species within the genus *Amblyceps* are endemic to NE India [41] and inadequately described or frequently synonymised [11], [42]. The other database sequence of *A. apangi* (accession number DQ508066) and all the database sequences of *G. trilineatus*, *G. telchitta*, and *E. pusillus* were not conspecific with the respective query sequences, and remained below the congeneric threshold thereby revealed deep conspecific divergences. This indicated possible cases of independently evolving lineages of a species from different geographical location [20] or cryptic species with low divergence, or even recently-diverged overlooked species [23], [29]. As such, the identification of species under the genera, viz., *Glyptothorax, Erethistes*, and *Amblyceps* remained inconclusive due to inadequate and perplexing descriptions from conventional taxonomy. Hence, in order to develop a correct barcode reference library, there is a paramount need of extensive revision, combining morphological and DNA barcoding of the extant species under these genera.

Thus, it can be concluded that 21 species representing 84% of the studied catfish species were identified straightforward through DNA barcoding that reaffirmed the efficacy of the technique. The study resolved some cases of synonymy, clarified the range distribution and revealed the catfish diversity in NE India. Occurrence of *Pterocryptis indicus* in NE India was not evident in the study and hence holds to agree upon arguments on the doubtful status of this species [12]. However, remaining 16% of the studied species representing 3 genera remained inconclusive and warrant further evaluation. Few database sequences were observed to be bearing misidentified species caption among the species those possess either confusing morphological description or share crypticism. Given that the database is enriched with the multiple sequences for a target species and for the extant species within a target genus from the range of distribution, the species taxonomy would be rectified and assessment of biodiversity would be correct and easier.

## Supporting Information

Table S1 **Morphological taxonomic keys observed versus described.**
(DOC)

Table S2 **List of the studied species, GenBank accession numbers of the analyzed sequences, the geographical position, and IUCN status.**
(DOC)
