# Supplementary material for: Identification and Re-Evaluation of Freshwater Catfishes through DNA Barcoding
Source: PLoS One. 2012 Nov 15;7(11):e49950. doi: 10.1371/journal.pone.0049950 (PMC3499493; doi:10.1371/journal.pone.0049950)
Supplement: Table S1 — Morphological taxonomic keys observed versus described. (DOC) [file pone.0049950.s001.doc]

**Table S1.** Key taxonomic features of the studied species vis-à-vis described characters for them. Disputing features are highlighted in remarks. Species are arranged in family wise. Taxonomic descriptions follow the leading taxonomic guide books in India.

| **Observed features** | | | **Described features by** | | | | **Remarks** |
| --- | --- | --- | --- | --- | --- | --- | --- |
|  | | | Talwar and Jhingran (1991) [6] | | Jayaram (1999)  [26] | |  |
| Family **Bagridae** | | |  | |  | |  |
| Genus  *Sperata* | | | | Only two species have been reported from native of study site | | |  |
| Species | | | |  | |  |  |
| 1 | *S. aor* (n = 3) | | |  | |  |  |
|  | i. presence of interneural shield in between basal bone of dorsal fin and occipital bone is conspicuous (genus character) | | | Snout rounded; maxillary barbells extend to base of caudal fin or beyond vs. much shorter in *S. seenghala*; width of mouth less than half of head length vs. one-third in *S. seenghala*; adipose fin long, its base about two times in rayed dorsal fin | | Snout rounded vs. spatulate in *S. seenghala*, width of gape of mouth less than ½ head length vs. ⅓ in *S. seenghala*; caudal fin with 17 rays vs. 19 to 21 in *S. seenghala* | Length of maxillary barbels is though long in this species vs. much short in sister species *S. seenghala* but it is not so long as noted by Talwar and Jhingran (1991), again the width of gape of mouth reflects very subtle difference |
|  | ii. Snout round | | |  |  |  |  |
|  | iii. maxillary barbels extend to mid of anal fin | | |  |  |  |  |
|  | iv. width of gape of mouth 42 to 43% in head length | | |  |  |  |  |
|  | v. dorsal fin base shorter than adipose | | |  |  |  |  |
| Genus  *Mystus* | | | |  | |  |  |
| Species | | | |  | |  |  |
| 2 | *M. bleekeri* (n = 8) | | |  | |  |  |
|  | i. Adipose fin inserted immediately behind rayed dorsal fin | | | Occipital process reaching basal bone of dorsal fin; Adipose fin long; Maxillary barbels extend posteriorly to anal fin; Body with two light bands, one above and the other below lateral line. | | Occipital process reaching basal bone of dorsal fin; adipose dorsal fin commencing almost after rayed dorsal fin; maxillary barbels reach anal fin; interorbital width less than 3.0 (2.0 to 3.0) in head length. | Extension of maxillary barbels up to end of anal as well as presence of two longitudinal bands on body are very helpful to diagnose this species from other congeners, but the interorbital width in head length is not incongruent to Jayaram (1999) |
|  | ii. maxillary barbels extend up to tip of anal fin | | |  |  |  |  |
|  | iii. body with two longitudinal bands one each above and below lateral line | | |  |  |  |  |
|  | iv. interorbital width 3 to 3.5 in head length | | |  |  |  |  |
| 3 | *M. cavasius* (n = 4) | | |  | |  |  |
|  | i. Adipose fin as long as in *M. bleekeri* | | | Adipose dorsal fin long; median longitudinal groove on head extends to base of occipital process; a dark spot on base of caudal fin often present | | Maxillary barbels reach caudal fin base or beyond; interorbital width more than 3.0 (3.0 to 4.0) in head length; a dark spot at base of caudal fin; no bands on body | Only the interorbital width in head length is not incongruent to Jayaram (1999) |
|  | ii. maxillary barbels extend beyond the point of bifurcation of caudal fin lobes | | |  |  |  |  |
|  | iii. longitudinal groove reaches base of occiput | | |  |  |  |  |
|  | iv. interorbital width 3.5 to 4 in head length | | |  |  |  |  |
| 4 | *M. vittatus* (n = 7) | | |  | |  |  |
|  | i. Adipose fin short and inserted after an interspace behind rayed dorsal fin | | | Eye diameter 4.5 to 6 times in head length; pectoral fin with 9 soft rays vs. 6 in *M. montanus*; body marked with 3 or 4 longitudinal pale blue or dark brown or black bands above and below lateral line vs. one or two longitudinal light bands in *M. montanus* | | Eye diameter 4.5 to 6 times in head length; pectoral fin with 9 soft rays; body with 3 or 4 longitudinal colour bands above and below lateral line; a dark shoulder spot; no spot at base of caudal fin | All the observed characters match well with described characters |
|  | ii. longitudinal groove extends to base of occiput | | |  |  |  |  |
|  | iii. body with four longitudinal dark brown bands | | |  |  |  |  |
|  | iv. pectoral fin with 9 soft rays | | |  |  |  |  |
| Genus  *Hemibagrus* | | | |  | | |  |
| Species | | | |  | |  |  |
| 5 | *H. menoda* (n = 2) | | | Previously described under the genus *Mystus* | | |  |
|  | i. interneural shield absent | | | Occipital process not reaching basal bone of dorsal fin; median longitudinal groove on head reaches base of occipital process; depth of body 4.4 to 5 times in standard length; clusters of small vertical spots along anterior part of lateral line | | Same as Talwar and Jhingran (1991) | Generally looks similar with congeners of *Sperata* but distinct from them due to the absence of interneural shield. Further, this species is mostly alike to congeners of *Mystus* but bear distinctness due to the presence of cluster of vertical spots along anterior part of lateral line |
|  | ii. occipital process not reaching basal bone of dorsal fin | | |  |  |  |  |
|  | iii. median longitudinal groove on head reaches base of occiput | | |  |  |  |  |
|  | iv. cluster of vertical spots along anterior part of lateral line presents | | |  |  |  |  |
| Genus  *Rita* | | | | Only one species has been reported from native of study site | | |  |
| Species | | | |  | |  |  |
| 6 | *R. rita* (n = 1) | | |  | |  |  |
|  | i. Teeth on palate in two elliptical patches, fairly wide apart | | | Teeth on palate in two elliptical patches, fairly wide apart | | Teeth on palate in two elliptical patches | The species is identifiable due to the presence of palatine teeth in two elliptical patches vs. only single patch in *R. chrysea* and *R. pavimentatus*, and two pear-shaped patches in *R. kuturnee* |
|  | ii. Barbels three pairs, maxillary barbels extend posteriorly to operculum | | |  |  |  |  |
| Family **Schilbeidae** | | | |  | |  |  |
| Genus  *Eutropiichthys* | | | | Only two species have been reported from native of study site | | |  |
| Species | | | |  |  |  |  |
| 7 | *E. vacha* (n = 7) | | |  | |  | Most of the key features are difficult to diagnose in early life stage. The length of nasal barbels as a taxonomic key is confusing, while cleft of mouth only provides easy diagnosis of both the species, yet it has not been weighted by Jayaram (1999) |
|  | i. Palatine band of teeth wider than  maxillary band | | | Branchiostegal rays 11, cleft of mouth  extends nearly to posterior edge of orbit;  vomero-palatine band of teeth wider  than maxillary band; nasal barbels rarely  extend up to posterior border of head | | Teeth on palate in a band wider than  premaxillary band, nasal barbels reach  hind border of head or slightly beyond |  |
|  | ii. Nasal barbels reach beyond posterior  edge of orbit, and 58 to 59% in head length | | |  |  |  |  |
|  | iii. Mouth cleft extends to middle of orbit | | |  |  |  |  |
| 8 | *E. murius* (n = 3) | | |  | |  |  |
|  | i. palatine teeth band very narrower  than maxillary band | | | Branchiostegal rays 5; cleft of mouth extends to anterior edge of orbit; vomero-paltine band of teeth narrower than or just as wide as maxillary band; nasal  barbels extend slightly beyond posterior  edge of orbit | | Teeth on palate in a band narrower than  premaxillary band or just equal to, nasal barbels reach a short distance behind posterior edge of eyes |  |
|  | ii. Nasal barbels reach beyond posterior of orbit, and 65 to 66% in head length | | |  |  |  |  |
|  | iii. Mouth cleft not reaches anterior edge of orbit | | |  |  |  |  |
| Genus  *Clupisoma* | | | | Only one species has been reported from native of study site | | |  |
| Species | | | |  |  |  |  |
| 9 | *C. garua* (n = 1) | | |  | |  |  |
|  | i. Abdominal edge keeled between vent and pelvic fin | | | A small adipose fin generally present, but only in young. Maxillary barbels extend to base of pelvic fins (or in young to middle of pelvics). Anal fin rays 29 to 36 | | Abdominal edge keeled between pelvic and vent. Pectoral fins not reaching pelvic. Maxillary barbels just reaching pelvic fin. Anal fin with 29 to 36 rays. | The abdominal edge, whether keeled or not, if keeled then up to what extent, is the key feature to diagnose this species from its sister species. Yet, this feature is not weighted by Talwar and Jhingran (1991). Again, in its sister species *C. bastari*, pectoral fins also do not reach pelvics. |
|  | ii. Maxillary barbels extend little beyond the base of pelvic | | |  |  |  |  |
|  | iii. Anal fin rays 34 | | |  |  |  |  |
| Genus  *Ailia* | | | | Only one species has been reported from native of study site | | |  |
| Species | | | |  | |  |  |
| 10 | *A. coila* (n = 3) | | |  | |  |  |
|  | i. Rayed dorsal fin absent | | | Anal fin with 58 to 75 rays vs. 76 to 90 in *A. punctata*; pelvic fins present vs. absent in *A. punctata* | | Anal fin rays 58 to 75; ventral profile not pronouncedly arched; body colour silvery to dull brown without any black blotch on caudal fin base or along side of body | This genus is distinguishable due to the absence of dorsal fin, number of anal fin rays count is very important for species identification |
|  | ii. Anal fin rays 60 | | |  |  |  |  |
|  | iii. Pelvic fins very small | | |  |  |  |  |
| Family **Sisoridae** | | | |  | |  |  |
| Genus  *Glyptothorax* | | | |  | |  |  |
| Species | | | |  | |  |  |
| 11 | *G. telchitta* (n = 3) | | |  | |  |  |
|  | i. Adhesive apparatus on thorax longer than broad and without a central pit | | | Adhesive apparatus on thorax longer than broad and without a central pit; dorsal fin as high as depth of body; anal fin inserted opposite or anterior to origin of adipose fin; body plain devoid of coloured bands; occipital process not reaching basal bone of dorsal fin | | Adhesive apparatus on thorax longer than broad and without a central pit; occipital process not reaching basal bone of dorsal fin; body rough with granulations or tubercles; nostrils separated from the snout by a distance equal to eye diameter; caudal peduncle height 3.0 to 3.8 times in length | This species is distinguishable from other congeners except *G. gracile* and *G. platypogonoides* due to rough body. But in later two species the dorsal fin is distinctly higher than depth of body. However, the particular character of body plain as mentioned in favour of this species by Talwar and Jhingran (1991) is confusing |
|  | ii. occipital process not reaching basal bone of dorsal fin | | |  |  |  |  |
|  | iii. rayed dorsal fin nearer origin of adipose dorsal fin than tip of snout | | |  |  |  |  |
|  | iv. body rough with profuse granulations, caudal peduncle height 3.5 to 3.7 times in length | | |  |  |  |  |
|  | v. anal fin inserted anterior of adipose fin | | |  |  |  |  |
| 12 | *G. trilineatus* (n = 1) | | |  | |  |  |
|  | i. Rayed dorsal fin inserted midway between tip of snout and origin of adipose fin | | | Adhesive thoracic apparatus devoid of central pit; dorsal fin shorter or as high as depth of body; anal fin inserted opposite or anterior to origin of adipose fin; body with three longitudinal coloured bands | | Origin of rayed dorsal fin midway between tip of snout than origin of adipose dorsal fin; body depth 4.5 times in standard length; body with three longitudinal bands | This species is identifiable due to the presence of three longitudinal bands, and due to well similarity with reference keys |
|  | ii. adhesive apparatus longer than broad | | |  |  |  |  |
|  | iii. body with three conspicuous longitudinal bands | | |  |  |  |  |
|  | iv. anal fin inserted anterior to origin of adipose fin | | |  |  |  |  |
| 13 | *G. striatus* (n = 1) | | |  | |  |  |
|  | i. ventral surface of pectoral fins plaited | | | Occipital process distinctly separated from basal bone of dorsal fin; adhesive apparatus as long as broad; skin on head and body rough, coarsely granulated or tuberculated; Sucking apparatus formed by skin-folds, appear not only on chest between pectoral fins but also on lower surface of outer rays of paired fins | | Ventral surface of paired fins plaited, occipital process not reaching basal bone of dorsal fin; Adhesive apparatus as long as broad | This species is the only native species of *Glyptothorax* having ventral surface of paired fins plaited |
|  | ii. adhesive apparatus as long as broad | | |  |  |  |  |
|  | iii. Occipital process distinctly separated from basal bone of dorsal fin | | |  |  |  |  |
|  | iv. skin on head coarsely granulated | | |  |  |  |  |
| Genus  *Gagata* | | | |  | |  |  |
| Species | | | |  | |  |  |
| 14 | *G. sexualis* (n = 2) | | |  | |  |  |
|  | i. Base of mandibular barbels inserted on a transverse row at the same level (genus character) | | | Median longitudinal groove extends only to base of occipital process; isthmus narrow; distal parts of fins not dusky; maxillary barbels longer than head; pectoral fins with a filamentous prolongation | | Median longitudinal groove does not extend to full length of supraoccipital process, but only up to base; maxillary barbels longer than head; pectoral fins with a filamentous prolongation | The fishes belonging to genera *Gagata* and *Nangra* are very similar in morphology, the species *G. sexualis* is distinguishable from its close congeners by the presence of maxillary barbels longer than head length vs. shorter than head in *G. cenia* |
|  | ii. median longitudinal groove extends only to base of occipital process | | |  |  |  |  |
|  | iii. maxillary barbels longer than head | | |  |  |  |  |
|  | iv. pectoral fins with a filamentous prolongation | | |  |  |  |  |
| 15 | *G. cenia* (n = 6) | | |  | |  |  |
|  | i. Maxillary barbels shorter than head | | | Maxillary barbels shorter than head; pectoral fins without any filamentous prolongation | | Maxillary barbels shorter than head; snout much longer than eye; head 3.6 to 4.0 times in standard length; anal fin with 13 to 15 rays |  |
| Genus  *Sisor* | | | |  | |  |  |
| Species | | | | Only single species has been reported from this region | | |  |
| 16 | *S. rhabdophorus*  (n = 4) | | |  | |  |  |
|  | i. upper ray of caudal fin much prolonged | | | Body elongate with a long tapering tail, a series of bony plates from base of dorsal fin to base of caudal fin are present | | Gill opening small, adipose dorsal fin in the form of a spine, upper ray of caudal fin much prolonged | The species is well identifiable through morphological features |
|  | ii. adipose dorsal fin in the form of a spine | | |  | |  |  |
| Genus  *Bagarius* | | | |  | |  |  |
| Species | | | |  | |  |  |
| 17 | *B. bagarius* (n = 3) | | |  | |  |  |
|  | i. Pelvic fin originate anterior to a vertical line through base of last dorsal-fin ray | | | Pelvic fins inserted anterior to last dorsal finray, adipose fin origin slightly to markedly posterior to anal fi origin, pectoral fin rays 9 to 12, gill rakers 6 to 9 on first arch | | Pelvic fin origin anterior to a vertical line through base of last dorsal-fin ray vs. posterior in *B. yarrelli*, pectoral fin rays 9 to 12 vs. 11 to 14 in *B. yarrelli* | The observed characters well match with the described characters, hence the species was easily identified |
|  | ii. pectoral fin rays 9 | | |  |  |  |  |
| Family **Siluridae** | | | |  | |  |  |
| Genus  *Ompok* | | | |  | |  |  |
| Species | | | |  | |  |  |
| 18 | *O. pabo* (n = 2) | | |  | |  |  |
|  | i. Dorsal fin rays 4 (genus character); anal fin rays 67 | | | Maxillary barbels short, extend posteriorly no further than head; pelvic fin with 9 or 10 rays | | Caudal fin forked, lobes pointed; lower border of eye below level of the cleft of mouth; maxillary barbels shorter than head length, pelvic fin rays 9 or 10, anal fin rays 66 to 71 | This species is identifiable due to short maxillary barbels |
|  | ii. maxillary barbels shorter than head | | |  |  |  |  |
| 19 | *O. bimaculatus*  (n = 2) | | |  | |  |  |
|  | i. maxillary barbels very long and extend beyond the base of anal fin | | | Anal fin with 57 or 58 branched rays; maxillary barbels extend posteriorly to (or slightly beyond) anal fin base vs. only to middle or tip of pectoral fin in *O. pabda* | | Maxillary barbels longer than head; pelvic fin rays eight; pelvic fins not reaching anal fin origin vs. reaching in *O. pabda*; anal fin insertion 2.0 to 4.0 times eye diameter behind last dorsal fin ray | The comparison of length of maxillary barbels is very helpful to distinguish all the congeners. But, this character is not weighted by Jayaram (1999). Further, the pelvic fins in the study sample distinctly reach the anal fin base that is not in identical to description by Jayaram (1999) |
|  | ii. pelvic fins reach anal fin | | |  |  |  |  |
|  | iii. Anal fin with 60 branched rays | | |  |  |  |  |
| Genus  *Wallago* | | | |  | |  |  |
| Species | | | | Only single species has been recorded from the wide geography of Indian region | | |  |
| 20 | *W. attu* (n = 2) | | |  | |  |  |
|  |  | | | Eyes with free orbital rim, lying entirely above level of corner of mouth, not visible from underside of head, gape of mouth wide and very long, reaching beyond anterior margin of eye | | Gape of mouth very wide, extending beyond eyes posteriorly | The morphological features are distinguishable |
| Family **Clariidae** | | | |  | |  |  |
| Genus  *Clarius* | | | |  | |  |  |
| Species | | | |  | |  |  |
| 21 | *C. batrachus* (n = 2) | | |  | |  |  |
|  | Dorsal fin inserted at a distance 6.0 times in length of head | | | Dorsal fin inserted at a considerable distance from end of head, the distance 4.5 to 6.0 times in length of head vs. 2.1 to 3.5 times all other congeners | | Distance from dorsal fin base to base of occipital process 4.5 to 6.0 times in head length vs. 2.0 to 3.5 in all the other congeners | Only a single species described from NE India, This species is easily identifiable through morphological features |
| Family **Heteropneustidae** | | | |  | |  |  |
| Genus  *Heteropneustes* | | | |  | |  |  |
| Species | | | |  | |  |  |
| 22 | *H. fossilis* (n = 3) | | |  | |  |  |
|  | i. Anal fin separated from the caudal fin by a deep notch | | | Anal fin separated from the caudal fin by a deep notch vs. confluent with caudal fin in *H. microps*. Occipital process not extending base of caudal fin vs. reaching in *H. microps* | | Anal fin separated from the caudal fin by a deep notch vs. confluent with caudal fin in *H. microps*. Occipital process not reaching base of caudal fin vs. reaching in *H. microps* | Only single species is reported from NE India. This species is easily identifiable through morphological features |
|  | ii. Occipital process not reaching base of caudal fin | | |  |  |  |  |
| Family **Erethistidae** | | | |  | |  |  |
| Genus  *Erethistes* | | | | Only one species has been reported from native of the study area | | |  |
| Species | | | |  | |  |  |
| 23 | | *E. pussilus* (n = 2) | |  | |  |  |
|  | | i. Serrations along outer margins of pectoral spine divergent | | Pectoral spine with 9 to 12 divergent serrae on anterior edge; body with 4 or 5 rows of tubercles | | Dorsal spine distinctly serrated vs. smooth in *E. maesotensis*; pelvic fins not extending to anal fin; pectoral spine with 9 to 2 serrae. | The genus is only distinguishable from the sister genus due to the presence of divergent serrations along outer margin of pectoral spine vs. mixed serration types of antrose in proximal half and retrose along distal half of pectoral spine in *Erethistoides*. This character is very difficult to diagnose without microscopic help |
|  |  | ii. Dorsal spine distinctly serrated | |  |  |  |  |
|  |  | iii. pelvic fin not extending to anal fin | |  |  |  |  |
| Family **Amblycipitidae** | | | |  | |  |  |
| Genus *Amblyceps* | | | |  | |  |  |
| Species | | | |  | |  |  |
| 24 | *A. apangi* (n = 1) | | |  | |  |  |
|  | i. Adipose fin very closely placed with caudal fin | | | Not recorded | | Not recorded | Vishwanath and Linthoingambi (2007) [11] redescribed both the endemic *A. apangi* and *A. arunachalensis* being valid species. The former is characterised by the presence of adipose fin very closely placed appearing confluent with caudal fin vs. widely separated in *A. arunachalensis* and *A. mangois*. Secondly, pinnate-like rays absent vs. pinnate-like rays present on the anterior margins of several procurrent rays in *A. arunachalensis* and *A. mangois*. |
|  | ii. Pinnate-like rays absent | | |  | |  |  |
| Family **Olyridae** | | | |  | |  |  |
| Genus  *Olyra* | | | |  | |  |  |
| Species | | | |  | |  |  |
| 25 | *O. longicaudata*  (n = 1) | | |  | |  |  |
|  | i. upper lobe of caudal fin almost twice as long as lower | | | Caudal fin forked, pectoral fin with 4 to 6 soft rays, upper lobe of caudal fin about twice as long as lower lobe vs. the upper lobe is only slightly longer than lower lobe in *O. horai* | | Caudal fin forked, upper lobe of caudal fin almost twice as long as lower, body depth 9 to 11 times in standard length vs. 11 to 12 times in *O. kempi*, anal fin rays 18 to 23 vs. 17 or 18 in *O. kempi* | Talwar and Jhingran (1991) recorded only single species from NE India, while Jayaram (1999) recorded two species; our specimens are similar to the descriptions of *Olyra longicaudata* and different from the description of *O. kempi* with respect to anal fin rays count |
|  | ii. anal fin with 19 rays | | |  |  |  |  |
