# Supplementary material for: Identification and Re-Evaluation of Freshwater Catfishes through DNA Barcoding
Source: PLoS One. 2012 Nov 15;7(11):e49950. doi: 10.1371/journal.pone.0049950 (PMC3499493; doi:10.1371/journal.pone.0049950)
Supplement: Table S2 — List of the studied species, GenBank accession numbers of the analyzed sequences, the geographical position, and IUCN status. (DOC) [file pone.0049950.s002.doc]

**Table S2.** List of species included in the study mentioning COI GenBank accession numbers of individuals analyzed under each species. The dataset include 101 sequences for 27 species, of which 75 sequences for 25 species were developed, while 26 sequences of related taxa were acquired from NCBI GenBank.

| **Family** | **Studied species** | **COI GenBank**  **Accession No.** | **Geographical**  **Position** | **IUCN (www.iucnredlist.org)** | |
| --- | --- | --- | --- | --- | --- |
|  |  |  |  | **Status described** | **Distribution described** |
| **Bagridae** | 1. *Mystus bleekeri* | JN228943 | 24.43 N 93.40 E | Least concern | Bangladesh, India, Nepal and Pakistan. Brahmaputra-Ganges system, as well as the Indus and Mahanadi River drainages |
|  |  | JN228944 | 24.47 N 93.10 E |  |  |
|  |  | JN228945 | 24.45 N 92. 54 E |  |  |
|  |  | JN628898 | 24.45 N 92. 54 E |  |  |
|  |  | JN628899 | 24.50 N 92.45 E |  |  |
|  |  | JN628901 | 24.43 N 93.40 E |  |  |
|  |  | JN628904 | 24.57 N 92.45 E |  |  |
|  |  | JN628928 | 24.47 N 93.10 E |  |  |
|  | 2. *Mystus cavasius* | JN228946 | 24.50 N 92.45 E | Least concern | Bangladesh, India, Nepal and Pakistan (uncertain). Ganges, Brahmaputra, Mahanadi, Subarnarekhar and Godavari river drainages |
|  |  | JN228947 | 24.57 N 92.45 E |  |  |
|  |  | JN228948 | 24.53 N 92.44 E |  |  |
|  |  | JN628905 | 24.47 N 93.10 E |  |  |
|  | 3. *Mystus vittatus* | JN228949 | 24.44 N 92.53 E | Least concern | Bangladesh, India, Nepal, Pakistan and Sri Lanka. Inland and coastal rivers throughout the Indian subcontinent and Sri Lanka |
|  |  | JN228950 | 24.51 N 92.58 E |  |  |
|  |  | JN228951 | 24.34 N 92.57 E |  |  |
|  |  | JN228952 | 24.16 N 92.31 E |  |  |
|  |  | JN228953 | 24.38 N 92.36 E |  |  |
|  |  | JN628887 | 24.38 N 92.36 E |  |  |
|  |  | JN628888 | 24.47 N 93.10 E |  |  |
|  |  | DQ508093* | NA |  |  |
|  | 4. *Mystus horai* | FJ170791* | 26.52 N 80.54 E | No entries found | No entries found |
|  | 5. *Rita rita* | JN628918 | 24.16 N 92.31 E | Least concern | Bangladesh, India and Nepal. Ganga-Brahmaputra river basin |
|  |  | EU417794* | NA |  |  |
|  |  | EU417793* | NA |  |  |
|  |  | EU417792 | NA |  |  |
|  | 6. *Sperata aor* | JN628929 | 24.45 N 92. 54 E | Least concern | Bangladesh, India, Nepal, Myanmar and Pakistan |
|  |  | JN628920 | 24.47 N 93.10 E |  |  |
|  |  | JN628930 | 24.50 N 92.45 E |  |  |
|  |  | FJ170790* | 26.52 N 80.54 E |  |  |
|  | 7. *Hemigabrus menoda* | JN697599 | 24.16 N 92.31 E | Least concern | Bangladesh, India and Nepal. Brahmaputra, Ganges and Mahanadi river drainage |
|  |  | JN697600 | 24.38 N 92.36 E |  |  |
| **Schilbeidae** | 8. *Eutropiichthys*  *murius* | JN228954 | 24.47 N 93.10 E | Least concern | Bangladesh, India and Nepal. Mahanadi river in Bengal and Ganges and Brahmaputra river drainage in North and North-east India |
|  |  | JN228955 | 24.45 N 92. 54 E |  |  |
|  |  | JN228956 | 24.50 N 92.45 E |  |  |
|  | 9. *Eutropiichthys*  *vacha* | JN228957 | 24.53 N 92.44 E | Least concern | Bangladesh, India, Nepal and Pakistan (uncertain). Larger freshwater rivers of the Gangetic provinces |
|  |  | JN228958 | 24.44 N 92.53 E |  |  |
|  |  | JN228959 | 24.51 N 92.58 E |  |  |
|  |  | JN228960 | 24.44 N 92.53 E |  |  |
|  |  | JN228961 | 24.51 N 92.58 E |  |  |
|  |  | JN228962 | 24.34 N 92.57 E |  |  |
|  |  | JN628877 | 24.43 N 93.40 E |  |  |
|  | 10. *Ailia coila* | JN628885 | 24.34 N 92.57 E | Near Threatened | Bangladesh, India, Nepal and Pakistan. Confined to Jamuna, Ganga, Brahmaputra and Mahanadi in India, Indus plains in Pakistan, Bangladesh and Nepal. |
|  |  | JN628886 | 24.16 N 92.31 E |  |  |
|  |  | JN815282 | 24.46 N 93.01 E |  |  |
|  |  | JN815284 | 24.46 N 93.01 E |  |  |
|  | 11. C*lupisoma garua* | JN628921 | 24.45 N 92. 54 E | Least concern | Bangladesh, India, Nepal and Pakistan. Rivers of Gangetic province, Mahanadi and Indus river drainage |
| **Sisoridae** | 12. *Bagarius bagarius* | JN697601 | 24.43 N 93.40 E | Near Threatened | Bangladesh, Bhutan, India and Nepal. Mekong, Chao Phraya, Ganges and Brahmaputra river drainages. |
|  |  | JN697602 | 24.47 N 93.10 E |  |  |
|  |  | JN815268 | 24.50 N 92.45 E |  |  |
|  |  | EU417766* | NA |  |  |
|  | 13. *Bagarius yarrelli* | DQ508069* | NA | Near Threatened | Bangladesh, China (Yunnan), India and Nepal. uncertain distribution- Cambodia, Indonesia, Lao People's Democratic Republic, Malaysia, Myanmar, Pakistan, Thailand and Viet Nam. |
|  |  | EU490855* | NA |  |  |
|  | 14. *Gagata cenia* | JN628891 | 24.53 N 92.44 E | Least concern | Bangladesh, India, Nepal and Pakistan. Mahanadi, Indus, Ganges and Brahmaputra river drainages. |
|  |  | JN628892 | 24.44 N 92.53 E |  |  |
|  |  | JN628893 | 24.51 N 92.58 E |  |  |
|  |  | JN628906 | 24.45 N 92. 54 E |  |  |
|  |  | JN628907 | 24.50 N 92.45 E |  |  |
|  |  | JN628925 | 24.47 N 93.10 E |  |  |
|  | 15. *Gagata sexualis* | JN628896 | 24.34 N 92.57 E | Least concern | Bangladesh, India and Nepal. Ganges and Brahmaputra river drainages. |
|  |  | JN628897 | 24.47 N 93.10 E |  |  |
|  |  | DQ846703* | NA |  |  |
|  | 16. *Glyptothorax telchitta* | JN628900 | 24.53 N 92.44 E | Least concern | Bangladesh, India, Nepal and Pakistan. Ganges and Brahmaputra river drainages. |
|  |  | JN628914 | 24.47 N 93.10 E |  |  |
|  |  | JN628915 | 24.45 N 92. 54 E |  |  |
|  |  | DQ514362* | NA |  |  |
|  | 17. G*lyptothorax striatus* | JN628902 | 24.47 N 93.10 E | Near Threatened | India and Bangladesh (uncertain). Restricted to Meghalaya of Northeast India. |
|  |  | DQ514361* | NA |  |  |
|  | 18. *Glyptothorax trilineatus* | JN628908 | 24.53 N 92.44 E | Least concern | China, India, Lao People's Democratic Republic, Myanmar, Nepal and Thailand |
|  |  | DQ508077* | NA |  |  |
|  | 19. *Sisor rabdophorus* | JN628916 | 24.50 N 92.45 E | Least concern | India and Bangladesh (uncertain). Lower Ganges river drainage |
|  |  | JN628917 | 24.57 N 92.45 E |  |  |
|  |  | JN628883 | 24.44 N 92.53 E |  |  |
|  |  | JN628884 | 24.51 N 92.58 E |  |  |
| **Siluridae** | 20. *Ompok bimaculatus* | JN628878 | 24.47 N 93.10 E | Near Threatened | Bangladesh, India, Pakistan, Sri Lanka and Myanmar (uncertain). |
|  |  | JN628919 | 24.38 N 92.36 E |  |  |
|  |  | FJ230073* | 24.57 N 88.06 E |  |  |
|  |  | FJ230074* | 24.57 N 88.06 E |  |  |
|  | 21. *Ompok pabo* | JN628879 | 24.45 N 92. 54 E | Near Threatened | Bangladesh, India, Myanmar (uncertain) and Pakistan (uncertain). Brahmaputra, Ganges and Jamuna river basins |
|  |  | JN628926 | 24.47 N 93.10 E |  |  |
|  |  | FJ230037* | 24.57 N 88.06 E |  |  |
|  |  | FJ230038* | 24.57 N 88.06 E |  |  |
|  | 22. *Wallago attu* | JN628923 | 24.53 N 92.44 E | Near Threatened | Bangladesh, India, Indonesia (Jawa), Myanmar, Nepal, Pakistan, Sri Lanka, Thailand and Viet Nam |
|  |  | JN628895 | 24.51 N 92.58 E |  |  |
|  |  | FJ170771* | 26.51 N 80.55 E |  |  |
|  |  | FJ170770* | 26.51 N 80.55 E |  |  |
| **Clariidae** | 23. *Clarias batrachus* | JN628924 | 24.44 N 92.53 E | Least concern | Widely distributed in Asia. |
|  |  | JN628880 | 24.50 N 92.45 E |  |  |
|  |  | HQ654701* | 14.09 N 120.06 E |  |  |
|  |  | GQ466402* | NA |  |  |
| **Heteropneustidae** | 24. *Heteropneustes fossilis* | JN628922 | 24.50 N 92.45 E | Least concern | South and Southeast Asia: Pakistan, India, Sri Lanka, Nepal, Bangladesh, Myanmar, Thailand and Laos. |
|  |  | JN628881 | 24.57 N 92.45 E |  |  |
|  |  | JN628882 | 24.53 N 92.44 E |  |  |
|  |  | GQ466398* | NA |  |  |
| **Erethistidae** | 25. *Erethistes pusillus* | JN628913 | 24.43 N 93.40 E | Least concern | Bangladesh, India and Nepal. Ganges and Brahmaputra river drainages |
|  |  | JN628894 | 24.44 N 92.53 E |  |  |
|  |  | DQ508074* | NA |  |  |
|  |  | DQ508079* | NA |  |  |
| **Amblycipitidae** | 26. *Amblyceps apangi* | JN628881 | 24.45 N 92. 54 E | Least concern | India. Brahmaputra River drainage in West Bengal, Nagaland and Arunachal Pradesh |
|  |  | DQ508066* | NA |  |  |
|  |  | EU490873* | NA |  |  |
| **Olyridae** | 27. *Olyra longicaudata* | JN697598 | 24.34 N 92.57 E | Least concern | India, Myanmar and Thailand. Brahmaputra River drainage in India, river drainages in Myanmar and the Mae Khlong River drainage in western Thailand. |

- ‘*’ denotes “sequences acquired from NCBI”
- ‘NA’ denotes “Not available”
